# Atomically Sharp Interface in an h-BN-epitaxial graphene van der Waals Heterostructure

**DOI:** 10.1038/srep16465

**Published:** 2015-11-20

**Authors:** Haikel Sediri, Debora Pierucci, Mahdi Hajlaoui, Hugo Henck, Gilles Patriarche, Yannick J. Dappe, Sheng Yuan, Bérangère Toury, Rachid Belkhou, Mathieu G. Silly, Fausto Sirotti, Mohamed Boutchich, Abdelkarim Ouerghi

**Affiliations:** 1 Laboratoire de Photonique et de Nanostructures (LPN), CNRS, Université Paris-Saclay, route de Nozay, F-91460 Marcoussis, France; 2 Synchrotron-SOLEIL, Saint-Aubin, BP48, F91192 Gif sur Yvette Cedex, France; 3 SPEC, CEA, CNRS, Université Paris Saclay, CEA Saclay, 91191, Gif-Sur-Yvette, France; 4 Laboratoire des Multimatériaux et Interfaces, UMR CNRS 5615, Université Lyon I, Université de Lyon, France; 5 GeePs, CNRS UMR8507, CentraleSupelec, Univ Paris-Sud, Sorbonne Universités-UPMC Univ Paris 06, 11 rue Joliot-Curie, Plateau de Moulon, 91192 Gif-sur-Yvette Cedex, France

## Abstract

Stacking various two-dimensional atomic crystals is a feasible approach to creating unique multilayered van der Waals heterostructures with tailored properties. Herein for the first time, we present a controlled preparation of large-area h-BN/graphene heterostructures via a simple chemical deposition of h-BN layers on epitaxial graphene/SiC(0001). Van der Waals forces, which are responsible for the cohesion of the multilayer system, give rise to an abrupt interface without interdiffusion between graphene and h-BN, as shown by X-ray Photoemission Spectroscopy (XPS) and direct observation using scanning and High-Resolution Transmission Electron Microscopy (STEM/HRTEM). The electronic properties of graphene, such as the Dirac cone, remain intact and no significant charge transfer i.e. doping, is observed. These results are supported by Density Functional Theory (DFT) calculations. We demonstrate that the h-BN capped graphene allows the fabrication of vdW heterostructures without altering the electronic properties of graphene.

Low-dimensional materials have been emerging recently as new systems with unique structure and size-dependent properties. While having lateral dimensions typically at the micron scale, the thickness of layers is at the atomic scale, rendering them “true” nanoscale materials with significant anisotropic properties. While graphene is undoubtedly the archetypical 2D material, inorganic layers derived from layered transition metal oxides or dichalchogenides have the potential to increase as well as diversify the portfolio of 2D systems available to date^1^.

Hexagonal boron nitride (h-BN) promises to be an ideal dielectric substrate for improved graphene-based devices^2^,^3^. h-BN is an insulating isomorph of graphite with boron and nitrogen atoms occupying the inequivalent A and B sublattices in the Bernal stacking structure. The different onsite energies of the B and N atoms lead to a large (5.97 eV) band gap and a small (1.7%) lattice mismatch with graphite^4^,^5^. h-BN is relatively inert, owing to the strong, in-plane, ionic bonding of the planar hexagonal lattice structure. The dielectric properties of h-BN compare favorably with silicon dioxide (SiO_2_), allowing the use of h-BN as an alternative gate dielectric. Moreover, the surface optical phonon modes of h-BN have energies double that of similar modes in SiO_2_, offering the possibility of improving the high-temperature and high-electric field performances of h-BN/graphene based devices compared to the usual oxide/graphene counterparts.

h-BN has proved to be an excellent substrate for monolayer graphene by improving the performance of the quantum yield, for example^2^. Thus, h-BN, produced on a large scale and possibly in an integrated process with graphene, is a strong candidate for new van der Waals heterostructures. Several routes are currently being pursued for creating epitaxial 2D materials on graphene layers^6^,^7^,^8^,^9^. Among these, the epitaxial approach based on SiC graphitization is a viable method for a large-scale graphene production^10^. A considerable advantage of this technique lies in the fact that wide-band gap semiconductor SiC wafers can be employed as a substrate, so that a graphene transfer step is not required. This allows the epitaxial graphene to be used as a substrate and h-BN as a top gate dielectric in h-BN/graphene based devices. The advantage is that layered h-BN/graphene heterostructures can be fabricated without the need of an adhesion layer, allowing the intrinsic properties of the graphene channel to be maintained, reducing scattering from impurities and charged traps associated with graphene/dielectric interfaces. However, to our knowledge, only two studies by Lin *et al.* have so far focused on 2D materials (MoS_2,_ WSe_2_ and h-BN) on epitaxial graphene/SiC^11^,^12^. The authors grew MoS_2_ on epitaxial graphene by CVD and performed TEM and Raman spectroscopy to investigate the interface of this heterostructure. Very recently, Yuan *et al.*^13^ have developed an easy approach to produce self-standing h-BN few- layers and monolayers using the Polymer Derived Ceramics (PDCs) method, the latter being easily dispersed in an alcoholic solution without the need of any further polymeric or surfactant stabilizers. This approach makes it possible to assemble h-BN with epitaxial graphene, paving the way for the creation of a well-ordered h-BN/graphene structure, providing opportunities for a wide spectrum of applications.

Here, we study the interface properties of h-BN on epitaxial graphene on 4H-SiC(0001). We demonstrate that the h-BN flakes can be deposited on the graphene in various sizes. Due to the inertness of both surfaces, van der Waals forces play a major role in the h-BN layer organization. Two distributions were observed using micro-Raman mapping: i) isolated h-BN thin flakes of 2×2 μm^2^ and ii) a continuous h-BN layer on the graphene surface. The h-BN/graphene heterostructure was also studied by Scanning and High-Resolution Transmission Electron Microscopy (STEM/HRTEM), Near Edge X-Ray Absorption Fine Structure (NEXAFS) and X-ray as well as angle-resolved X-ray Photoemission Spectroscopy (XPS and ARPES). Measurements of electronic structure were corroborated by Density Functional Theory (DFT) calculations.

## Results and Discussion

**Figure 1:**
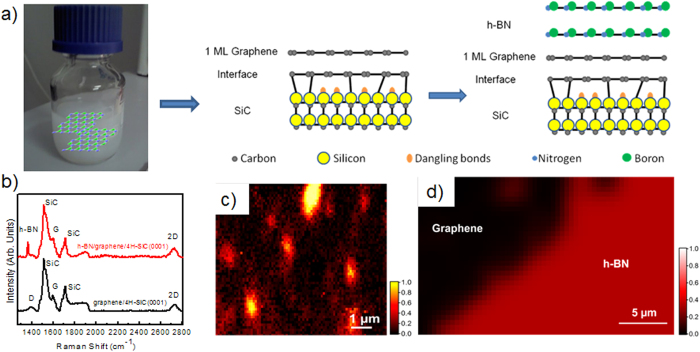
h-BN flakes on graphene/SiC(0001) substrates. (**a**) Schematic illustration of our method for growing h-BN/graphene/SiC. After ultrasonication of the h-BN suspension a few drops of the solution were deposited on epitaxial graphene. (**b**) Comparison of micro-Raman spectra taken on the pristine graphene (black line) and h-BN/graphene (red line) layers. Integrated intensity Raman map of the h-BN E_2g_ vibration mode for two representative areas of the h-BN/graphene heterostructure (**c**) area with small h-BN flakes, (**d**) uniform layers and an abrupt interface between h-BN and the graphene substrate.

**Figure 2:**
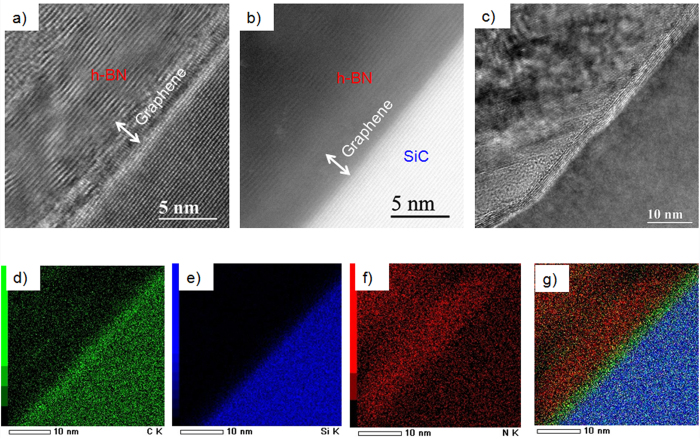
(**a**) Bright-field Scanning Transmission Electron Microscopy (STEM) image and (**b**) corresponding High Angle Annular Dark Field (HAADF) STEM image of h-BN on graphene/4H-SiC heterostructure, (**c**) High-Resolution Transmission Electron Microscopy (HRTEM) image of h-BN on the step edges of graphene on SiC, (**d**–**f**) EDX elemental maps showing, respectively, the spatial distribution of C, Si and N in the selected area of the h-BN on graphene/4H-SiC heterostructure. The overlay of the three maps is given in (**g**).

Thermal decomposition of undoped 4H-SiC(0001) can be used to produce large graphene films with a long-range order and high electron mobility^14^. Monolayer, bilayer, and few-layer graphene on SiC can be obtained by tuning the different experimental parameters (temperature, annealing times, and thickness of SiC substrate)^15^. The graphene used in this study was obtained by annealing 4H-SiC at 1550 °C in 800 mbar argon for 10 min [see Methods]. The h-BN layers were synthesized using the Polymer Derived Ceramics method by adding lithium nitride (Li_3_N) micropowders to liquid-state polyborazylene (PBN)^13^. Incorporating Li_3_N as a crystallization promoter allows h-BN to crystallise at a lower temperature (1200°C) than under classical conditions (1800 °C)^16^. The synthesized h-BN layers were then dispersed in ethanol with a low concentration in order to avoid a huge agglomeration of flakes and to control their dispersal over epitaxial graphene. Figure 1(a) presents a schematic illustration of the steps required for the fabrication of the h-BN/graphene structure. After the growth step, the graphene sample was cooled down to room temperature and transfered *ex situ* from the growth chamber. After ultrasonication of the h-BN suspension, a few drops of solution were deposited on the epitaxial graphene. Raman spectroscopy was carried out to characterize the morphology of h-BN on graphene. Figure 1(b) compares the micro-Raman spectra of pristine graphene (black line) and the h-BN/graphene heterostructure (red line) in the wavenumber range of 1300–2800 cm^−1^. Several intense peaks are visible in this range; these correspond to second-order Raman bands originating in the SiC substrate^17^. In the pristine sample, the graphene contributions are also observed and identified by three main structures: i) the D band at 1370 cm^−1^, ii) the G band at 1595 cm^−1^ and iii) the 2D band at 2720 cm^−1^; the small D peak indicates the high quality of the sample. In the case of h-BN/graphene, we note a new peak at ∼1367 cm^−1^ originating from the E_2g_ mode of h- BN vibration^18^,^19^,^20^. In Fig. 1(c,d), we observe the integrated intensity Raman map of the h-BN E_2g_ vibration mode for two representative areas of the h-BN/graphene heterostructure. As it is shown by the different colour scale in Fig. 1(c), this area presents small h-BN flakes with a different width (~1–2 μm). On the other hand, we can also observe the coalescence of various flakes into uniform layers and an abrupt interface between h-BN and the graphene substrate (Fig. 1(d)). As the Raman analysis shows, with the use of the drop-casting method it was not possible to control the thickness of the h-BN flakes and their organization on the epitaxial graphene substrate. However, without the need of a particular treatment, where the h-BN/graphene heterostrucutre was formed, the interface was really abrupt without any interdiffusion. In order to investigate in more detail this interface between the h-BN and epitaxial graphene, STEM/HRTEM experiments were performed. A cross-sectional view was chosen along the (11–20) SiC zone axis. STEM images revealed the thickness of the graphene layers and the detailed crystalline structure of both the h-BN and the graphene (Fig. 2(a), Bright-field STEM and (b) High angle annular dark field (HAADF) STEM). As observed from representative STEM images of our heterostructure, the graphene grown on SiC, produced here, was predominantly composed of few layers. The graphitic layers were atomically flat and showed a continuous film at the step edges. It is obvious from Fig. 2(a), that multilayer stacking of h-BN occurred on the few layer graphene. The measured interlayer distance of about 0.344 ± 0.002 nm, was characteristic of interplanar spacing in h-BN (002) planes^21^,^22^. As can be seen in HRTEM of Fig. 2(c), h-BN multilayers were not perfectly flat on the graphene substrate all over the sample. In different areas, we found that the h-BN layers lay predominately with a misorientation angle following the (11–20) nanofacets of the SiC substrate. Using Energy-Dispersive X-ray spectroscopy (EDX) we mapped the elemental distribution of C, Si and N in a selected area of the h-BN on graphene/4H-SiC heterostructure (Fig. 2(d–f)). Indeed, a sharp vertical interface between the h-BN and graphene layers was visible. The intensity of the nitrogen maps across the interface also showed that the sharpness of the graphene/h-BN interface was within 1 nm, corresponding to the spatial resolution of the EDX elemental mapping (due to the thickness sample and the convergence of the electron probe) (Fig. 2(g)). Moreover, from the EDX signal coming from specific spots on the sample we also obtained a quantitative analysis (using the SiC substrate as reference). The average N/B ratio for the whole h-BN region was estimated to be 1.1 ± 0.1, confirming the stoichiometry of the h-BN layer.

**Figure 3:**
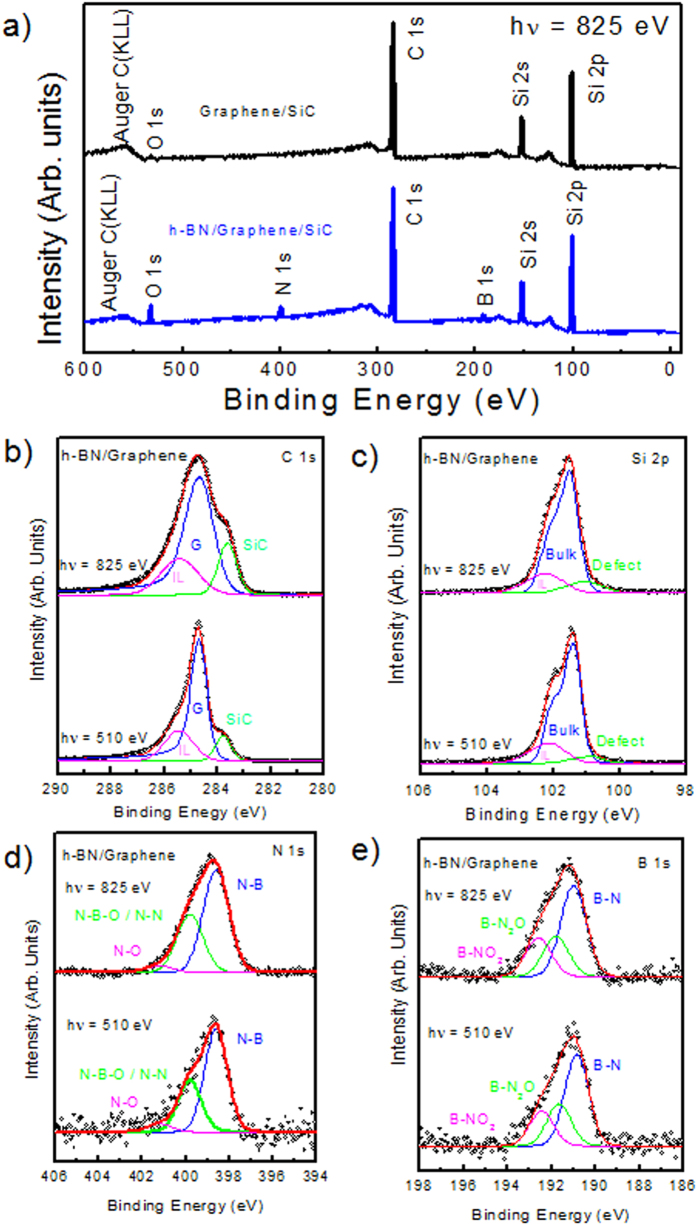
(**a**) XPS overview spectra at hν= 825 eV of the pristine graphene (black line) and the h-BN/ graphene heterostrucuture (blue line). High resolution XPS spectra of h-BN/graphene for (**b**) C 1s core level, (**c**) Si-2p core level (**d**,**e**) B 1*s* and N 1*s* core levels at hν = 825 eV (bulk sensitive, top panel) and hν=510 eV (surface sensitive, bottom panel).

**Figure 4:**
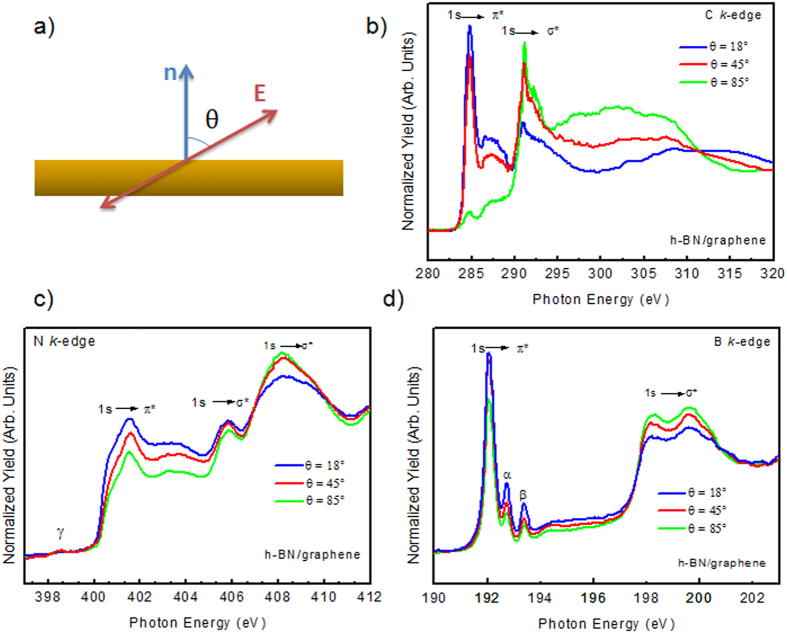
(**a**) Schematic representation of the NEXAFS geometry: θ represents the angle between the polarization vector 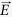 and the surface normal 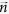. (**b**) carbon (**c**) nitrogen and (**d**) boron K-edge, NEXAFS spectra of h-BN/graphene, measured for different incidence angles of the linear polarized synchrotron light. (θ = 18°, 45° and 85°).

To investigate the atomic composition, as well as the chemical bonding environment of our samples, XPS measurements were carried out for the pristine graphene and the h-BN/graphene heterostructure, as shown in Fig. 3. The XPS measurements performed over a wide energy range (hν = 825 eV) showed that the C 1*s*, Si 2*s*, and Si 2*p* peak intensities were not notably affected by the h-BN deposition (Fig. 3(a)), whose presence was confirmed by the B 1*s* and N 1*s* core level peaks observed on the h-BN/graphene spectrum. The different contributions to the spectra were extracted by a curve fitting procedure (see Methods). The depth position of the corresponding species across the h-BN/graphene interface was identified by varying the incident photon energy (hv = 510 and 825 eV) and thus changing the surface and bulk sensitivity. The experimental data points are displayed as dots. The solid line is the envelope of the fitted components. The C 1*s* and Si 2*p* spectra of the h-BN graphene heterostructure are shown in Fig. 3(b,c), respectively. The C1*s* spectra show the conventional deconvolution expected for epitaxial graphene on SiC(0001)^23^,^24^,^25^, characterized by three contributions attributed to the substrate (SiC) at BE = 283.8 eV, the graphene layer (G) at BE = 284.6 eV and the interface layer at 285.3 eV. No evidence of C-B bond at 283. 2 eV^26^,^27^ or C-N and C-O bonds expected at higher BE, 286.5 eV^28^ and 286.2 eV^29^, respectively, were present in the spectra. These observations confirm that there were no covalent bonds formed between graphene and h-BN, nor graphene contamination. The Si 2*p* doublet was reconstituted with a 2*p*^*1/2*^:2*p*^*3/2*^ ratio of 0.5 and a spin-orbit splitting of 0.6 eV^30^. Both spectra consisted of a main Si 2*p* peak at 101.5 eV. The small shoulder at 102.1 eV was attributed to an interface layer area of the SiC(0001) substrate, while the component at 100.9 eV was attributed to the presence of Si clusters formed when Si-C bonds were broken during graphitization^31^,^32^. No additional components related to Si-O bonds^29^ or Si-N-C (101.9 eV^28^) were present confirming the formation of a perfect heterostructure between graphene and h-BN, with no evidence of interdiffusion or contamination. XPS spectra of B 1*s* and N 1*s* are shown in Fig. 3(c,d), respectively, for two different photon energies (hν = 825 eV and hν = 510 eV). The B1*s* and N1*s* spectra exhibited three peaks fitted with Gaussian components (full width at half-maximum, *fwhm* ~ 1.4 eV). The main peak at binding energy BE ~ 191 eV for the B 1s and BE ~ 398.6 eV for the N 1*s* were attributed to B-N bonding, matching those reported for bulk h-BN^33^,^34^,^35^,^36^. The boundaries between h-BN flakes could present a higher density of defects, which could also provide a preferential site for adsorbates (e.g. oxygen). As expected from the first-principle calculations of Orellana *et al.*^37^, the most stable defects in h-BN are the self-interstitial N_i_ and the nitrogen vacancy. Interstitial nitrogen binds strongly with a N atom of the lattice forming a N-N pair^37^ and the substitution of a nitrogen atom with oxygen is preferred to the substitution of a boron atom because of the closer electronegativity between oxygen and nitrogen atoms. According to the core-level shift model proposed by Caretti *et al.*^38^, suggesting a core level shift per O substituent N of ~0.8 eV (toward the higher BE) we could attribute the peaks at BE ~ 191.8 eV and 192.6 eV in the B 1s spectra to B-N_2_O and B-NO_2_, respectively, (one and two N vacancies are replaced by one and two oxygen). In the case of N 1*s,* the components at higher BE ~ 399.8 eV and ~401.2 were attributed to O-B-N^39^ or N-N^40^ and N-O^40^,^41^, respectively. Unoccupied electronic states of the h-BN/graphene heterostructure were also probed by NEXAFS spectroscopy. The C 1*s*, B 1*s* and N 1*s* NEXAFS spectra were measured for different incidence angles of the linear polarized synchrotron light. As shown in Fig. 4(a), we indicate with θ the angle between the polarization vector 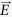 and the surface normal 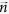 (θ = 18°, 45° and 85°). Figure 4(b) shows the C 1*s* NEXAFS spectra. These spectra were characterized by two sharp resonances at 285.3 eV and 291.7 eV and a broad structure at 292.7 eV. These features were characteristic of pristine graphene on SiC^24^,^42^, and were assigned to π^*^, 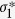 and 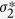 resonance, respectively. For the grazing incidence of light, when the direction of polarization vector 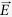 was close to the surface normal (θ = 18°), the π^*^ resonance intensity was strongly enhanced. This feature decreased with an increased incident angle and was almost suppressed for normal incidence (θ = 85°). As expected for a planar π conjugated system such as graphene, the σ^*^ resonances revealed the opposite angular dependence behaviour. The sharpness of the NEXAFS features indicated a well-defined bonding environment and a long-range periodic order in the electronic structure. Thus, the NEXAFS spectra unequivocally proved the conservation of *sp*^2^ bonds between carbon atoms in the h-BN/graphene heterostructure. The fact that no modifications were induced on the C 1*s* NEXAFS spectra with respect to pristine graphene, confirmed the dominant role of van der Waals interactions between graphene and h-BN layers. Figure 4(c,d) show the B 1*s* and N 1*s* NEXAFS spectra. In addition to the characteristic B(1*s* → π^*^) excitonic transition of h-BN at ~192.0 eV (which is a specific fingerprint of *sp*^2^- hybridized B atoms in the hexagonal h-BN network, *i.e.*, a trigonal B-N_3_ bonding environment) and two σ^*^ peaks at ~198.2 eV and ~199.5 eV, two other excitonic π^*^ peaks were observed in the B 1*s* x-ray absorption edge at ~192.7 eV and ~193.3 eV^38^. These peaks, labelled in this study α and β, were assigned to B surrounded by 1 and 2 N-void defects, respectively. Peaks α and β were due to an intermediate situation between B-N_3_ and B-O_3_ environments, *i.e*., B-N_2_-O and B-NO_2_, respectively^38^, confirming the photoemission results. The N 1*s* NEXAFS spectra displayed an excitonic peak with a maximum centred at ~401.5 eV and two σ^*^ transitions at ~405.8 eV and ~408.1 eV, as found in the literature for a bulk h-BN^43^,^44^. Moreover, theoretical calculations predicted an empty level induced by neutral N_i_ at around 0.6 eV above the valence band maximum (VBM) of h-BN, *i.e.* just above 397 eV for the 4 eV energy gap of h-BN^37^,^43^. Then the small resonance (γ in Fig. 4(c)) observed at about 398.6 eV can be related to this particular defect^45^, which was also clearly identified in the N 1*s* XPS spectra. For the orientation of the h-BN flakes on graphene we clearly saw that the flakes were not all perfectly flat on the graphene substrate, as observed in the HRTEM image. In fact, for normal incidence of light and a polarization vector almost in the plane of the sample (θ = 85°), the π^*^ resonance was still visible, even if attenuated with respect to the grazing incidence (θ = 18°).

**Figure 5:**
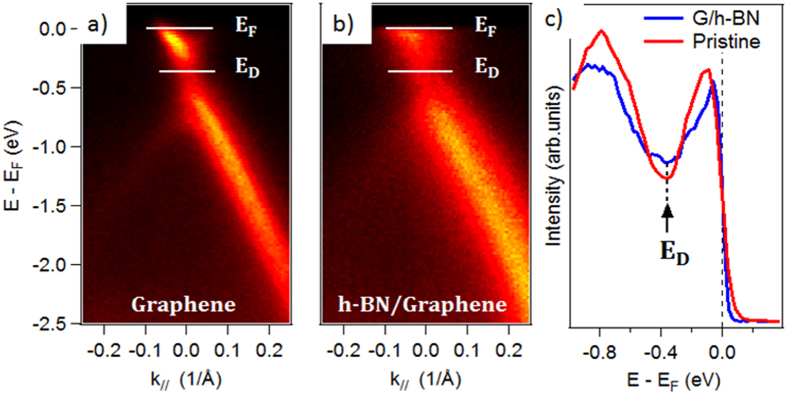
(**a**,**b**) ARPES measurements of pristine graphene and h-BN/graphene, measured at hν = 60 eV, through the K-point, in the ΓK direction; (**c**) ARPES intensity integrated spectra as a function of the binding energy, extracted from the 2D ARPES map, for the initial pristine graphene (red line) and h-BN/graphene (blue line).

**Figure 6:**
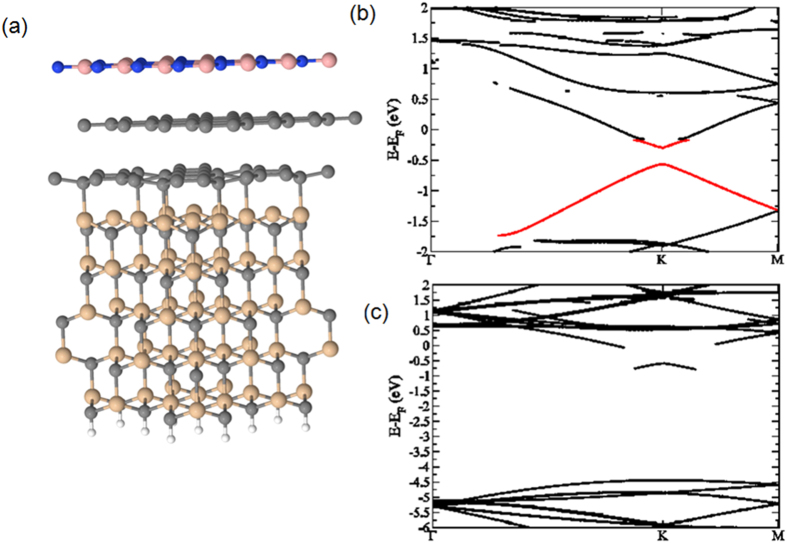
(**a**) DFT and vdW optimised geometrical structure of the h-BN/graphene/ZLG/SiC van der Waals heterostructure, (**b**) graphene bandstructure and (**c**) h-BN bandstructure.

In order to clearly highlight the effect of h-BN on graphene’s electronic structure, ARPES measurements were carried out. Figure 5 shows the valence band structure around the *K* point of the first graphene Brillouin zone, for the pristine graphene layer and the h-BN/graphene heterostructure. On the pristine graphene layer (Fig. 5(a)), the linear dispersion of the *π* band can be observed. As it has already been observed many times in the case of epitaxial graphene with the Si termination, the *π* bands formed a cone, for which the *π* branches crossed at the Dirac point (*E*_*D*_) at −0.3 eV below the Fermi level (*E*_*F*_). This *n*-type doping is typical of epitaxial graphene and is due to the charge transfer from the SiC substrate. After h-BN deposition (Fig. 5(b)), these *π* branches were still clearly defined, proving that the growth of h-BN did not affect the 2D structure of the graphene. The Dirac point was located at −0.3 eV below the Fermi level, demonstrating the absence of electronic transfer between h-BN and graphene layers, corroborating the absence of an h-BN doped graphene layer. The full width at half maximum (*fwhm*) of the Dirac cone branches probably increased because of the interaction between the graphene and h-BN layers. It has been proposed that a bandgap would be induced in graphene aligned in a commensurate way to a h-BN substrate^46^. To confirm these features, we performed DFT calculations on the h-BN/graphene van der Waals heterostructure (see Methods). We considered here a commensurate h-BN monolayer on top of a graphene/SiC(0001) substrate (Fig. 6(a)). Structural determinations yielded an h-BN-graphene distance of 3.3 Å with a binding energy of 77 meV/atom, and a graphene-buffer layer of 3 Å, with a binding energy of 166 meV/atom, in agreement with the expected values for such systems. Our DFT calculations confirmed that, in this case, a gap of about ~315 meV is opened at the K point (Fig. 6(b)). This gap opening was due to the electrostatic interaction between the h-BN and the graphene planes, breaking the symmetry of the graphene lattice. Moreover, as shown in (Fig. 6(c)), h-BN maintained its insulating properties (calculated band gap of ~5.5 eV). In the present case, as the experimental data showed no gap openings for graphene, this suggested the two following hypotheses: (i) the h-BN monolayer was lying with a rotated structure with respect to the graphene plane, preventing from symmetry breaking (ii) several layers of h-BN were present on graphene, the interaction between the BN planes reducing the electrostatic interaction between h-BN and graphene, which forbids the gap opening.

## Conclusions

In summary, we are able to synthesize a high-quality multilayer h-BN/graphene heterostructure. The h-BN is synthesized by using the PDCs route, from a liquid-state polyborazylene as polymeric precursor additivated with lithium nitride (Li_3_N) micropowders in order to obtain highly crystallized material. The epitaxial h-BN/graphene domains were shown by micro- Raman spectroscopy. More importantly, the STEM/HRTEM results showed that the h-BN layers are highly crystalline without any interdiffusion. XPS and ARPES spectroscopy shows a sharp interface between graphene and h-BN layers. This demonstrates that epitaxial graphene may be a suitable template to combine with alternative vdW solids due to its lack of dangling bonds, chemical inertness, and ability to remain intact under high stress. This study demonstrates a simple and efficient route for the controlled fabrication of multilayered graphene/h-BN heterostructures with promising applications in nanoelectronics.

## Methods

The h-BN on graphene/4H-SiC(0001) (Si-face, 4° off towards [11–20]) (Si-face) was created in two stages: first few-layer graphene was produced on a 4H-SiC(0001) commercial wafer off-axis. The sample was heated to 1100 °C in a UHV (10^−5^ mbar) and then further heated to 1550 °C in an Ar atmosphere for 10 mn. It was then cooled down to room temperature and transferred *ex-situ* from the growth chamber to the analysis chamber. The h-BN layers were synthesized using the Polymer Derived Ceramics route by adding lithium nitride (Li_3_N) micropowders to liquid-state polyborazylene (PBN), as recently described^13^. The synthesized h-BN layers were then dispersed in ethanol and a few drops of the latter solution were deposited on the epitaxial graphene. Cross-sectional TEM samples were prepared using a focused ion beam (FIB). Protective layers of amorphous carbon were deposited on the sample, initially with the electron beam to avoid surface damage. The HRTEM/STEM studies were done on a Jeol 2200FS microscope working at 200 kV and equipped with an aberration corrector for the probe (STEM mode) and an Ultra-High Resolution (UHR) pole piece.

XPS/ARPES experiments were carried out on the TEMPO beamline^47^ (SOLEIL French synchrotron facility) at low temperature (~120 K). The HU80 Apple II undulator was set to deliver linearly polarized light. The photon energy wes selected using a high-resolution plane grating monochromator, with a resolving power E/ΔE that can reach 15000 over the whole energy range (45–1500 eV). During the XPS measurements, the photoelectrons were detected at 0° from the sample surface normal 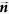 and at 46° from the polarization vector 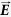. A Shirley background was subtracted in all core level spectra. The C 1*s* spectra were fitted by a sum of a Gaussian function convoluted with a Doniach-Sunjic lineshape. An asymmetry factor α was used, where α = 0.1 eV (peak G) and α = 0 eV (SiC an IL). The graphene thickness was calculated from the ratio between the intensity of the G and SiC components^48^,^49^ extracted from XPS data using the following equation: 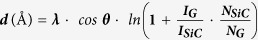. For a photon energy of 510 eV we had an escape depth λ of 7.2 Å. In the geometry of our XPS experiment the take-off angle θ was 0° and ***I***_***G***_ and ***I***_***SiC***_ represented the area of the G and SiC components of the C 1s peak extracted from the deconvolution. For epitaxial graphene on SiC, the areal density of the C atom in graphene ***N***_***G***_ was about three times that of C atoms in SiC^49^. We obtained a thickness ***d*** = 14.9 Å corresponding to about 6 ML of graphene. The Si 2*p* spectra were fitted by sums of Voigt curves, that is, the convolution of a Gaussian (of full-width at half-maximum GW) by a Lorentzian (of full-width at half-maximum LW). Where the LW are fixed at 45 meV^50^. In the case of B 1*s* and N 1*s,* the peaks were fitted by the sum of Gaussian functions (full width at half-maximum, *fwhm* ~ 1.4 eV). For ARPES measurements, the photon energy (h*ν* = 60 eV) and sample orientation were set in order to explore the k-space region around the K point in the ΓK direction of the Brillouin zone. The spot size was 100 × 40 (H × V) μm.

*Ab initio* calculations to describe the electronic structure of the h-BN/graphene van der Waals heterostructure were performed using a very efficient DFT localized orbital molecular dynamic technique (FIREBALL)^51^. Basis sets of *sp*^3^*d*^5^ for B, *sp*^3^ for C, N and Si, and *s* for H were used with cutoff radii (in atomic units) *s* = 4.5, *p* = 4.9, *d* = 5.2 (B), *s* = 4.5, *p* = 4.5 (C), *s* = 4.2, *p* = 4.2 (N), *s* = 4.8, *p* = 5.4 (Si) and *s* = 4.1 (H)^52^. In this study we considered a supercell of 5 ML SiC(0001), with a zero-layer graphene and an AB stacked graphene plane on top. The lateral size roughly corresponded to a 4×4 unit cell of graphene. The bottom layer was saturated with hydrogen atoms. On top of the supercell, we set an h-BN monolayer. The geometry of the system was then relaxed at 0 K, using a sample of 32 k-points in the Brillouin zone, maintaining the last three bottom layers in bulk positions^53^. The final distance between the 2D material planes was determined using the well-known LCAO-S + vdW formalism^54^, which specifically takes into account van der Waals interaction in the frame of DFT. A set of 300 special k points along the Γ–K-M path was used for the band structure calculations on the optimized geometry. Finally, a gap correction of 1.18 was applied, in order to correct the well-known DFT deficiency and to get a better agreement with the experiments, following the method proposed by Park *et al.*^55^.

## Additional Information

**How to cite this article**: Sediri, H. *et al.* Atomically Sharp Interface in an h-BN-epitaxial graphene van der Waals Heterostructure. *Sci. Rep.* **5**, 16465; doi: 10.1038/srep16465 (2015).
